# In Vitro Bacterial Contamination of Amniotic Fluid: Effects on Fluorescence Polarization Lung Maturity Testing

**DOI:** 10.1155/S1064744995000408

**Published:** 1995

**Authors:** R. Phillip Heine, Susan Harding, Pegi Emmett, Edward Ashwood, Roger R. Lenke

**Affiliations:** 1 Department of Obstetrics and Gynecology University of Colorado Denver CO USA; 2 Department of Clinical Pharmacology University of Colorado Denver CO USA; 3 Department of Pathology University of Utah School of Medicine Salt Lake UT USA; 4 Magee-Womens Research Institute 204 Craft Avenue Pittsburgh PA 15213 USA

## Abstract

*Objective:* We sought to determine the effect of bacteria on fluorescence polarization (FPOL) testing of amniotic fluid.

*Methods:* * Fusobacterium necrophorum* and *Escherichia coli* were inoculated at concentrations of 10^3^ and 10^6^/ml in amniotic-fluid specimens from 4 patients with no clinical or laboratory evidence of infection. The FPOL results were obtained at inoculation and again at 24 h of incubation. The results were compared using analysis of variance (ANOVA).

*Results:* The FPOL results from inoculated specimens were all within 2% of the uninoculated controls. The specimens incubated with bacteria showed a < 1–19% variation when compared with the time-zero uninoculated controls. However, uninoculated controls incubated for 24 h exhibited a 2–12% variation when compared with the time-zero controls, suggesting that the variation present was not secondary to the bacterial co-incubation.

*Conclusions:* In vitro, neither bacterial inoculation nor prolonged co-incubation influences FPOL results beyond the effect of incubation alone. FPOL appears to be an appropriate test to assess fetal lung maturity in patients in whom intraamniotic infection is a concern.


Infectious Diseases in Obstetrics and Gynecology 3:98–101 (1995)
© 1995 Wiley-Liss, Inc.

### In Vitro Bacterial Contamination of Amniotic Fluid: Effects on Fluorescence Polarization Lung Maturity Testing

**R. Phillip Heine, Susan Harding, Pegi Emmett, Edward Ashwood, and Roger R. Lenke**

*Departments of Obstetrics and Gynecology (R.P.H., S.H., R.R.L.) and Clinical Pharmacology (P.E.), University of Colorado, Denver, CO, and Department of Pathology (E.A.), University of Utah School of Medicine, Salt Lake City, UT*

Abstract

***Objective:*** **We sought to determine the effect of bacteria on fluorescence polarization (FPOL) testing of amniotic fluid.**

***Methods: Fusobacterium necrophorum*** **and** ***Escherichia coli*** **were inoculated at concentrations of $10^3$ and $10^6$/ml in amniotic-fluid specimens from 4 patients with no clinical or laboratory evidence of infection. The FPOL results were obtained at inoculation and again at 24 h of incubation. The results were compared using analysis of variance (ANOVA).**

***Results:*** **The FPOL results from inoculated specimens were all within 2% of the uninoculated controls. The specimens incubated with bacteria showed a < 1–19% variation when compared with the time-zero uninoculated controls. However, uninoculated controls incubated for 24 h exhibited a 2–12% variation when compared with the time-zero controls, suggesting that the variation present was not secondary to the bacterial co-incubation.**

***Conclusions:*** **In vitro, neither bacterial inoculation nor prolonged co-incubation influences FPOL results beyond the effect of incubation alone. FPOL appears to be an appropriate test to assess fetal lung maturity in patients in whom intraamniotic infection is a concern.** © 1995 Wiley-Liss, Inc.

Key words

Intraamniotic infection, prematurity, pregnancy, *Escherichia coli*, *Fusobacterium necrophorum*

Idiopathic preterm labor is complicated by bacterial colonization of the amniotic fluid in 0–24% of cases.[1] It is conceivable that bacterial contamination from an occult infection could adversely affect the results of fetal lung maturity testing. The only previous study addressing this issue has shown that a vaginal isolate of *Escherichia coli* was able to produce phosphatidyl glycerol, which caused a false-positive result in a vaginal-pool specimen.[2] Because amniocentesis is used to diagnose occult bacterial infection as well as assess fetal lung maturity in patients presenting with preterm labor or preterm premature rupture of the fetal membranes, further research is needed to address the effects of bacteria on amniotic-fluid maturity testing.

In 1976, Shinitsky et al.[3] first published an article showing the relationship of fluorescence polarization (FPOL) to fetal lung maturity. Recent modifications of this test have allowed its introduction to clinical practice for both routine and high-risk obstetric indications.[4,5] No studies have addressed the effect of bacteria on FPOL results;

**Address correspondence/reprint requests to Dr. R. Phillip Heine, Magee-Womens Research Institute, 204 Craft Avenue, Pittsburgh, PA 15213.**

Clinical Study

Received May 22, 1995
Accepted July 25, 1995

therefore, we sought to determine whether in vitro bacterial inoculation as well as 24-h bacterial co-incubation would affect the FPOL results.

### MATERIALS AND METHODS

### Amniotic-Fluid Collection and FPOL Assay

We obtained amniotic-fluid specimens from 4 women receiving prenatal care at University Hospital between August 1992 and August 1993. All 4 participants were in the third trimester of pregnancy (between 28 and 38 weeks of gestation) at the time the amniotic fluid was collected by transabdominal amniocentesis. Three of the participants were being evaluated for intrauterine growth retardation and one of the participants was being evaluated for preterm labor. All had intact membranes. There was no evidence of intraamniotic infection, as shown by Gram's stain, glucose evaluation, and subsequent amniotic-fluid culture. An initial FPOL was determined on the fresh amniotic-fluid specimen, as previously described.[4,5] The 4 specimens were specifically chosen because of their FPOL values (very mature, very immature, borderline, or barely mature). Our laboratory FPOL values are as follows: mature, $\leq 260$; immature, $\geq 290$; and borderline, $> 260$ but $< 290$.

### Amniotic-Fluid Inoculation and Incubation

The amniotic fluid was stored at −80°C and thawed to room temperature on the day of experimentation. The specimens were inoculated with either *E. coli* (EC), stock no. ATCC 12014, or *Fusobacterium necrophorum* (FN), stock no. ATCC 25286. Both organisms are known intraamniotic pathogens.[6] The EC were prepared by inoculation in thioglycolate broth with subsequent incubation at 35°C for 8 h. The organisms were harvested by centrifugation, washed 3 times in sterile phosphate-buffered saline (PBS), and diluted in PBS to a final concentration of $1 \times 10^4$ and $1 \times 10^7$ CFU/ml, as determined by spectrophotometer and verified plate count. The FN were obtained from bacteria cultured for 48 h on CDC anaerobic agar. The bacteria were then transferred to a reduced enriched thioglycolate broth and incubated anaerobically for 14 h until maximum turbidity was achieved. The bacteria were harvested by centrifugation and washed 3 times in reduced PBS. The washed cells were resuspended in reduced sterile PBS to a final concentration of $1 \times 10^4$ and $1 \times 10^7$ CFU/ml.

The maternal amniotic-fluid specimens were divided into 1-ml samples, and 100 μl of the varying bacterial suspensions were added. The FPOL results were obtained at time zero (inoculation) and again at 24 h (incubation). There were totals of 5 specimens at time zero (saline control, $10^3$ EC; $10^6$ EC; $10^3$ FN; $10^6$ FN) and 5 similar specimens at 24 h. The EC/amniotic-fluid samples were incubated aerobically and FN/amniotic-fluid samples anaerobically for 24 h at 35°C.

After incubation, FPOL analysis and bacterial colony counts were performed on each specimen. The EC were plated on McConkey agar, incubated for 48 h at 35°C, and counted. The FN were plated on anaerobic agar, incubated anaerobically for 72 h at 35°C, and counted.

### Statistical Analysis

The differences in FPOL results at time zero and at 24 h were ascertained using analysis of variance (ANOVA).

### RESULTS

Table 1 lists the FPOL and bacterial colony count results. At time zero, freshly run FPOL were all within 1% of the control (frozen) FPOL results. All inoculated specimens (16 aliquots) were within 2% of the control FPOL value at time zero. After incubation for 24 h, 10 of the 16 specimens inoculated with bacteria were within 5% of the time-zero control FPOL result, while 6 of the 16 specimens showed more than a 5% variation from the time-zero control value (range, 6–19%). This was not limited to the inoculated specimens, as the control specimens incubated at 35°C also showed wide variability, with values differing from 2 to 12% from the time-zero controls. The tendency was for a slight increase (less-mature results) in the FPOL values, as only 1 of the 16 inoculated specimens and 1 of 4 controls showed a decrease (more-mature values). The largest increases appeared to be in those specimens inoculated with the FN (2–19% vs. 0–6% EC). No results reached statistical significance when compared with the control FPOL.

The growth in amniotic fluid was dependent on the bacterium type. The anaerobe FN did not grow in the amniotic-fluid samples, while the aerobe EC exhibited an increase up to 4-fold in growth during the 24-h incubation.

**TABLE 1. Effect of in vitro bacterial inoculation and co-incubation on fluorescence polarization (FPOL) results[a]**

| | Control | $10^3$ EC | $10^6$ EC | $10^3$ FN | $10^6$ FN |
|---|---|---|---|---|---|
| Initial FPOL = 186 | | | | | |
| FPOL @ 0000 h | 186 | 187 | 187 | 187 | 187 |
| FPOL @ 2400 h | 209 (12) | 194 (4) | 198 (6) | 193 (4) | 182 (2) |
| CFU/ml @ 2400 | 0 | $5 \times 10^6$ | $9 \times 10^6$ | $<10$ | $<10$ |
| Initial FPOL = 250 | | | | | |
| FPOL @ 0000 h | 248 | 247 | 250 | 250 | 252 (1) |
| FPOL @ 2400 h | 238 (4) | 249 | 259 (4) | 262 (6) | 276 (11) |
| CFU/ml @ 2400 | 0 | $3 \times 10^7$ | $3 \times 10^7$ | 40 | $4 \times 10^4$ |
| Initial FPOL = 268 | | | | | |
| FPOL @ 0000 h | 270 | 270 | 270 | 270 | 271 |
| FPOL @ 2400 h | 288 (7) | 282 (4) | 273 (1) | 321 (19) | 317 (17) |
| CFU/ml @ 2400 | 0 | $10^7$ | $10^7$ | 50 | $5 \times 10^4$ |
| Initial FPOL = 330 | | | | | |
| FPOL @ 0000 h | 327 | 327 | 325 | 333 (2) | 333 (2) |
| FPOL @ 2400 h | 334 (2) | 208 (6) | 325 | 335 (2) | 338 (3) |
| CFU/ml @ 2400 | 0 | $6 \times 10^6$ | $6 \times 10^6$ | $<10$ | $10^4$ |

[a]Numbers in parenthese represent percent change from the control FPOL at 0000 h. Values without parentheses represent a difference of $<1\%$. EC, *Escherichia coli*; FN, *Fusobacterium necrophorum*.

### DISCUSSION

Our results show that neither bacterial inoculation of amniotic fluid nor prolonged bacterial co-incubation significantly affects the clinical interpretation of FPOL results. The amniotic-fluid specimens with varying initial FPOL values inoculated with bacteria did not exhibit any difference in FPOL values, regardless of the bacterial type or inoculum density. The variability observed in the specimens incubated in 24 h with EC or FN was also exhibited in the control specimens, suggesting that the bacterial inoculum was not responsible for the variation.

The FPOL results are based on the competition of albumin and surfactant for a fluorophore ligand.[7] Fluorescence will be high when the ligand is bound to albumin but low when bound to pulmonary surfactant. Surfactant degradation would, therefore, lead to a higher fluorescence value. We speculate that the variability in our study was secondary to the phospholipid degradation that occurs when specimens are kept at room temperature or warmer for prolonged periods.[8] Our results were consistent with this premise in that 14 of 16 values from the incubated specimens were greater (less mature) than the time-zero controls.

The bacterial growth in amniotic fluid was dependent on the bacterial type. All 4 amniotic-fluid specimens supported the aerobe EC, while the anaerobe FN exhibited declining colony counts over the 24-h incubation. No differences in FPOL results were seen with either bacterium at either the high or low colony counts. This suggests that the byproducts of actively growing or even lyzed bacteria will not adversely affect FPOL results.

Fluorescence polarization is a rapid, automated, reliable measure of fetal lung maturity. It appears from our current study that the coexistent bacterial contamination of amniotic fluid with *E. coli* or *F. necrophorum* will not influence FPOL results beyond the effect of incubation alone. Therefore, FPOL testing appears to be an appropriate diagnostic technique to assess fetal lung maturity in patients in whom occult amniotic-fluid infection is a concern. Subsequent work utilizing other bacterial species will further clarify this issue.

### REFERENCES

1. Armer TL, Duff P: Intraamniotic infection in patients with intact membranes and preterm labor. Obstet Gynecol Surv 46:589–593, 1991.
2. Schumacher RE, Parisi VM, Steady HM, Tsao FHC: Bacteria causing false positive test for phosophatidylglycerol in amniotic fluid. Am J Obstet Gynecol 1067–1068, 1985.
3. Shinitzky M, Goldfisher A, Bruck A, et al.: A new method for assessment of fetal lung maturity. Br J Obstet Gynaecol 83:838–844, 1976.
4. Hagen E, Link JC, Arias F: A comparison of the accuracy of the TDx-FLM assay, lecithin-sphingomyelin ratio, and phosphatidylglycerol in the prediction of neonatal respiratory distress syndrome. Obstet Gynecol 82:1004–1008, 1993.

5. Ruch AT, Lenke RR, Ashwood ER: Assessment of fetal lung maturity by fluorescence polarization in high-risk pregnancies. J Reprod Med 38:133–136, 1993.
6. Romero R, Sirtori M, Oyarzun E, et al.: Infection and labor. V. Prevalence, microbiology, and clinical significance of intraamniotic infection in women with preterm labor and intact membranes. Am J Obstet Gynecol 161: 817–824, 1989.
7. Ashwood ER, Chamberlain BV: Binding of fluorescent phosphatidylcholine in amniotic fluid. Obstet Gynecol 71: 370–374, 1988.
8. Schwartz DB, Engle MJ, Brown DJ, Farrell PM: The stability of phospholipids in amniotic fluid. Am J Obstet Gynecol 141:294–298, 1981.
